# Eigen-disfigurement model for simulating plausible facial disfigurement after reconstructive surgery

**DOI:** 10.1186/s12880-015-0050-7

**Published:** 2015-03-27

**Authors:** Juhun Lee, Michelle C Fingeret, Alan C Bovik, Gregory P Reece, Roman J Skoracki, Matthew M Hanasono, Mia K Markey

**Affiliations:** Department of Electrical and Computer Engineering, The University of Texas at Austin, 2501 Speedway, Stop C0803, Austin, TX 78712 USA; Department of Plastic Surgery, The University of Texas MD Anderson Cancer Center, 1515 Holcombe Blvd, Houston, TX 77030 USA; Department of Behavioral Science, The University of Texas MD Anderson Cancer Center, 1515 Holcombe Blvd, Houston, TX 77030 USA; Department of Biomedical Engineering, The University of Texas at Austin, 107 W Dean Keeton St, Stop C0800, Austin, TX 78712 USA; Department of Imaging Physics, The University of Texas MD Anderson Cancer Center, 1515 Holcombe Blvd, Houston, TX 77030 USA

**Keywords:** Facial disfigurement, Reconstructive surgery, 3D surface image, Simulation, Head and neck cancer

## Abstract

**Background:**

Patients with facial cancers can experience disfigurement as they may undergo considerable appearance changes from their illness and its treatment. Individuals with difficulties adjusting to facial cancer are concerned about how others perceive and evaluate their appearance. Therefore, it is important to understand how humans perceive disfigured faces. We describe a new strategy that allows simulation of surgically plausible facial disfigurement on a novel face for elucidating the human perception on facial disfigurement.

**Method:**

Longitudinal 3D facial images of patients (N = 17) with facial disfigurement due to cancer treatment were replicated using a facial mannequin model, by applying Thin-Plate Spline (TPS) warping and linear interpolation on the facial mannequin model in polar coordinates. Principal Component Analysis (PCA) was used to capture longitudinal structural and textural variations found within each patient with facial disfigurement arising from the treatment. We treated such variations as disfigurement. Each disfigurement was smoothly stitched on a healthy face by seeking a Poisson solution to guided interpolation using the gradient of the learned disfigurement as the guidance field vector. The modeling technique was quantitatively evaluated. In addition, panel ratings of experienced medical professionals on the plausibility of simulation were used to evaluate the proposed disfigurement model.

**Results:**

The algorithm reproduced the given face effectively using a facial mannequin model with less than 4.4 *mm* maximum error for the validation fiducial points that were not used for the processing. Panel ratings of experienced medical professionals on the plausibility of simulation showed that the disfigurement model (especially for peripheral disfigurement) yielded predictions comparable to the real disfigurements.

**Conclusions:**

The modeling technique of this study is able to capture facial disfigurements and its simulation represents plausible outcomes of reconstructive surgery for facial cancers. Thus, our technique can be used to study human perception on facial disfigurement.

**Electronic supplementary material:**

The online version of this article (doi:10.1186/s12880-015-0050-7) contains supplementary material, which is available to authorized users.

## Background

Patients with facial cancers are at particular risk for experiencing disfigurement as they may undergo considerable appearance changes from their illness and its treatment. Individuals undergoing facial reconstruction often have extensive tumors requiring radical surgical ablation of the primary site, and are, therefore, at heightened risk for experiencing facial disfigurement and functional impairment.

Increasing attention is being given to evaluating the psychosocial consequences of facial disfigurement, particularly for patients with head and neck cancers. Although individual reactions to disfigurement can vary considerably, body image difficulties are well documented among patients with head and neck cancer [1-3]. Many of these patients report feeling discounted or stigmatized due to their appearance following surgical treatment [4]. Disfigurement related to head and neck cancer has also been associated with worsened relationship with partners, impaired sexuality, depression, social isolation, and anxiety [5-8].

Individuals with difficulties adjusting to facial cancer are clearly concerned about how others perceive and evaluate their appearance [9]. However, there is a significant gap in knowledge regarding how others *actually* perceive and process disfigured faces. Information about the threshold at which disfigurement is noticeable and which aspects of disfigurement are most salient would benefit patients and healthcare providers alike. These data could be used to inform psychological interventions that help patients with facial disfigurement gain a more accurate understanding of how they are perceived in society, which has a strong potential to facilitate their psychosocial adjustment.

The best way to study the human perception of facial disfigurements is to show patients with facial disfigurement to human observers directly, and asking them to answer how they perceive the disfigurements. However, it is not feasible to recruit real patients for such an observer study. An alternative way is showing the observers 2D/3D photographs or videos of patients with facial disfigurement. However, such approaches possess critical weakness; we cannot control the degree and location of facial disfigurement.

Therefore, it is crucial to have a mathematical model to simulate facial disfigurement resulting from facial cancer treatments. This will allow us to control the degree and location of facial disfigurement, while removing the effect of the natural variability in facial morphology. For example, some patients may have more noticeable disfigurement than others, even if they underwent the same reconstructive procedure. Since we cannot control these variations, it is evident that they will add uncertainty to any model of the human perception of facial disfigurement. Using a mathematical model to create realistic simulations of disfigurement will enable control over the location and level of disfigurement. Moreover, such a model will make it possible to apply the same disfigurement to the faces of people of different ages and genders.

Simulating surgical outcomes on the human face has been extensively studied. In the field of computer-assisted surgery, its main focus has been on simulating the possible changes that arise from craniofacial surgery using volumetric reconstruction of patients’ CT data and/or 3D surface facial images. Most previous studies have tried to estimate soft tissue changes after the correction (such as osteotomy) of bony parts of the face [10-16] by using modeling techniques, including physics based models such as the Finite Element Model (FEM).

Within the field of plastic surgery, much effort has been expended toward predicting the outcomes of facial aesthetic surgery. For example, many algorithms have been proposed to predict outcomes of rhinoplasty by using computer graphic and image processing techniques on the patients’ 3D surface facial images or 3D rendering of volumetric reconstructions of their CT images [17-21].

Recently, Bottino et al. [22] introduced a simulation tool for facial aesthetic surgery. In their work, once a 3D surface facial image with a selected target region (e.g. nose, chin, mouth) for the aesthetic surgery is submitted, their system searches the *k* most similar faces in their face database using the entire face area except the target region. Then the facial target regions of the *k* most similar faces suggested by the system as well as their average are used to morph the original target region of the patient. They evaluated their system using panel ratings of laypersons and reported that the simulation with the mathematically averaged facial target region obtained the best panel attractiveness rating for most of their simulation cases. In addition, Claes et al. [23] recently introduced a simulation method to objectively assess the discordance of a given face of oral and maxillofacial surgery patients. In their method, a face space was constructed from 3D surface facial images of normal controls using Principal Component Analysis (PCA). Similar to the work of Bottino et al. [22], they utilized the normal (unaffected) part of a patient’s face to search a synthetic face from the face space. The resulting synthetic face can be seen as the face of patient’s identical twin without facial abnormality, which can be directly compared to the patient’s face to assess his/her facial abnormality for planning appropriate surgical actions.

However, no prior studies considered the facial disfigurement that remains after reconstructive surgery. From the results of previous work, there exists a limitation on helping patients who have to live with permanent facial disfigurement. This implies a significant need for developing a modeling strategy such as our disfigurement modeling technique.

Moreover, previous studies do not account for any textural appearance changes that arise from surgical treatment. This is because prior methods focus on overall structural changes, and not on any disfigurement remaining after the surgery. However, some reconstructive surgeries on patients with facial cancer (e.g., reconstruction of the orbit using his/her own tissue) can entirely change the textural appearance of the face. Hence, modeling strategies that can incorporate textural aspects of disfigurement are also worthy of study and implementation.

Here we present a new strategy that enables realistic modeling of the types of disfigurement that persist following facial cancer treatment and reconstructive surgery. Our approach employs 3D surface facial images of patients with facial disfigurement. This tool can be applied to other faces to provide control of the location and degree of disfigurement. We utilize PCA to capture longitudinal structural and textural variations found within each patient with facial disfigurement over the treatment. We treat such variations as disfigurement. Each disfigurement is smoothly stitched on a healthy face by seeking a Poisson solution to guided interpolation using the gradient of the learned disfigurement as the guidance field vector. To show the usefulness of the proposed disfigurement model, we quantitatively evaluated the modeling technique and also conducted an observer study using experienced medical professionals in which they evaluated the appearances of the simulated facial disfigurement.

## Methods

### Dataset: disfigured faces

In order to develop surgically plausible models of facial disfigurement, it is crucial to have 3D facial images of patients who have had excisions of facial tumors and reconstruction of structures in the face. This study employed 3D facial images acquired using a 3dMDcranial System (3dMD, Atlanta, GA) under an IRB (Institutional Review Board) approved protocol of The University of Texas MD Anderson Cancer Center, Houston, Texas, USA (Protocol ID of 2009–0784). There exists a companion IRB protocol approved by The University of Texas at Austin, Austin, Texas, USA (Protocol ID of 2010-02-0027) for data analysis.

The dataset consists of 3D facial images of patients aged 18 or older who had facial cancer and underwent or were scheduled for reconstructive surgery at The University of Texas MD Anderson Cancer Center. Informed consent (written) was obtained from all patients who participated in this research study. Additional consent was obtained for their images to be published in scientific papers. The dataset included the pre-operative (viz., prior to reconstructive surgery) 3D facial images and up to 4 post-operative 3D images (after initial reconstructive surgery) of patients’ faces obtained at 1, 3, 6, and 12 month(s) post reconstruction clinic appointments. These images were used to study the different types of facial disfigurement and their structural and textural changes over time.

To date, a total of 150 patients were recruited to the ongoing study. To learn structural and textural changes over time due to the reconstruction process, we utilized images of patients who had completed pre-op and at least 3 post-op visits (i.e., any three of 1, 3, 6, and 12 month post-op visits) (N = 72) to develop a model to simulate disfigurement on other faces. Among those patients, we removed any patients whose 3D images showed no visible disfigurement (N = 31), who did not have their 3D facial images taken (N = 8), or whose 3D images contained substantial artifacts introduced by problems in the acquisition process (e.g., calibration errors) (N = 16). After that, a total of 17 patients (3 females and 14 males, 79 images in total) were included in this analysis. Their ages ranged from 50 to 83 (mean: 64). Among 17 patients, 7 patients had visible disfigurement in their mid-face area only (eye, nose, or mouth area), while 10 patients had visible disfigurement in the periphery (forehead, cheek, chin, or neck area). We tabulate the information regarding each disfigured face region, the disease characteristics, and its location for those patients in Table 1 (Reconstruction procedure details for each patient are tabulated in Additional file 1).

**Table 1:** **Disease characteristics and location of disfigurement on the faces**

	**Patient ID**	**Disfigured region**	**# of images**	**Histology**	**Disease site**
Periphery	P1	M, LC, LN	5	SCC	Oral cavity, mandible
	P2	RC, RN, LN	5	SCC	Oral cavity
	P3	LC, LN	5	SCC	Cheek
	P4	FH, LC	5	Sarcoma	Forehead/Scalp
	P5	M, LC, LN	5	SCC	Mandible
	P6	M, RC, RN	4	SCC	Mandible
	P7	RC, RN	5	SCC	Ear
	P8	M, RC, RN	4	SCC	Oral cavity
	P9	M	5	SCC	Oral cavity
	P10	M, LC, RC, LN, RN	4	SCC	Oral cavity, mandible
Mid-Face	M1	FH, LE, N, RE	4	SCC	Orbit
	M2	N, M, RC	5	SCC	Maxilla
	M3	RE, N, RC	5	BCC	Orbit
	M4	N, LE, LC	5	Sarcoma	Nose
	M5	LE, LC	5	Sarcoma	Maxilla
	M6	LE	4	ACC	Maxilla
	M7	N	4	Melanoma	Nose

*Abbreviations*: *FH* Forehead, *LE* Left Eye, *N* Nose, *RE* Right Eye, *LC* Left Cheek, *M* Mouth, *RC* Right Cheek, *LN* Left Neck, *RN* Right Neck, *SCC* Squamous Cell Carcinoma, *BCC* Basal Cell Carcinoma, *ACC* Adenoid Cystic Carcinoma.

**Figure 1:**
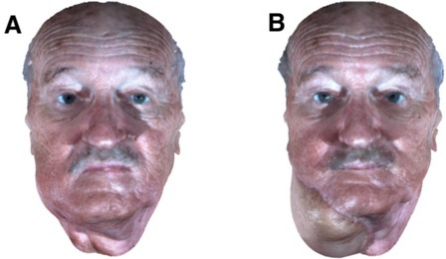
**3D facial images of one patient.** Example pre-operative **(A)** and post-operative **(B)** 3D facial images of one patient who underwent right neck composite resection followed by reconstructive surgery using the anterolateral thigh free flap.

All 3D images were cropped to remove unnecessary regions (e.g., clothes and back of the head) when developing the facial disfigurement models. The number of vertices in the 3D images after cropping ranged from 50,000 to 70,000. Although such number of vertices is enough to show the morphology of the face, it is not enough to adequately capture the texture. There is still a lack of texture detail when we rendered the face interpolating the color information at each vertex. To solve this problem, we increased the resolution of 3D images by subdividing the 3D images linearly. Each triangle was divided into 4 triangles using a new vertex that is linearly interpolated. Color information (RGB) at the newly identified vertices was extracted from the corresponding location of the original 2D texture image. The final number of vertices after the subdivision process ranged from 150,000 to 200,000. Figure 1 depicts an example of pre- and post-operative 3D facial images of a patient who underwent oncologic and reconstructive surgery.

### Dataset: non-disfigured faces

The surgically plausible disfigurement models are added to 3D facial images of non-disfigured individuals to evaluate the quality of the model. We used the Binghamton University 3D Facial Expression (BU-3DFE) Database as a source of non-disfigured individuals [24]. It is a publically available 3D face database of 3D facial images acquired using the 3dMDface system manufactured by 3dMD (Atlanta, GA). With the agreement of the technology transfer office of the SUNY at Binghamton, the database is available for use by external parties [25]. Analysis of this kind of dataset does not meet the definition of human subjects research and does not require IRB review at The University of Texas at Austin. As BU-3DFE database is a publicly available resource there was no need to obtain consent for their faces to be published in scientific papers.

The BU-3DFE database consists of 2500 3D facial expression models of 100 adult human subjects. The database contains 56 female and 44 male subjects, ranging age from 18 to 70 years, and includes the major ethnic groups White, Black, East-Asian, Middle-east Asian, Indian, and Hispanic Latino. Each subject performed seven different expressions which are neutral, happiness, disgust, fear, angry, surprise, and sadness, all captured using the 3dMD face system. Among the available 2500 facial images, we utilized only the raw 3D images (i.e., without cropping) of neutral expression faces. A total of 91 raw 3D images were used after removing 9 images having a missing neck area. Just as with the dataset of disfigured faces, all 91 images were cropped to remove unnecessary regions and their resolution linearly increased to 150,000 – 200,000 vertices.

### Preprocessing

#### Establishing full correspondence of examples

**Figure 2:**
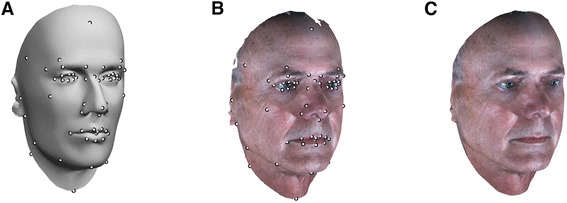
**Establishing full correspondence between samples.** A total 61 fiducial points (white dots) are used to establish full correspondences between samples. The fiducial points are manually annotated on both a 3D mannequin facial model **(A)** and a 3D facial image of a patient **(B)**. After completing all correspondence steps, his original 3D face was fully reproduced using the 3D mannequin facial model **(C)**. Note that the algorithm fills any holes on the original 3D facial image of the patient.

In order to model both structural and textural disfigurements, it is necessary to establish full correspondence of all faces. This is a difficult problem as: 1) each face has a different number of vertices and 2) 3D images obtained from the 3dMD system contain various types of noise, such as holes (missing data). The 3dMD system projects a random speckle pattern on the face, and uses that pattern to create the 3D images of subjects using triangulation. Oily areas of the face (e.g., foreheads or cheeks) or facial hair (e.g., mustaches) often result in reflecting the speckle pattern from the 3dMD system. As a result, holes remain in such areas since there is no pattern to match by triangulation. To solve these issues and to achieve a good correspondence between all of the faces, a mannequin facial model was used (Figure 2A). This facial model was treated as a reference that was warped to reproduce each patient’s facial morphology. This is similar to the seminal work of Cootes et al. [26], except the direction of modeling; they warped each 2D face image to the mean shape, while our method warps the reference to each 3D surface facial images. We set the number of vertices of the mannequin facial model to be 150,000. We placed denser vertices on the mid-face area than on peripheral areas since the mid-face has more complex structures than do peripheral areas. Note that there exist algorithms for establishing dense correspondences between healthy faces (e.g., [27,28]) as well as dysmorphic faces (e.g., [23,29-33]). Among those previous works for dysmorphic faces, some [23,29,30] utilized pre-computed spatially dense mask to establish the correspondence between the faces, while the others [28,31-33] used manually annotated fiducial points. The former can be a good alternative for our application. However, it has not been thoroughly validated for our patient samples. Thus, similar to the latter, we used the method described below to establish dense correspondence between the faces.

The first step taken was to manually annotate (by J.L.) a set of 61 fiducial points on the 3D surface images. The fiducial points used are shown in Figure 2A-B. The point set consists of: 1) 45 key fiducial points defined according to the rich literature on human facial anthropometry [34], for which there are established specifications of their locations, 2) 16 additional points outlining facial structures (e.g., eye, nose, and lips) and the entire facial boundary. It has been shown that most facial fiducial points can be identified reliably by human observers [35]. In practice, annotating these fiducial points for most faces can be done in approximately 5 minutes. After the annotation, we roughly aligned all faces (including the mannequin facial model) by translating the tip of the nose of each face to the point at (x y z) = (0, 0, 5) *cm*, to cause the centroid of the vertices of the face to be located near the origin.

The second step is to conform the size and location of the reference face model *M* to a given 3D surface image M^*^ using the Procrustes method [36]. The fiducial points of *M* and M^*^, *L,* and L^*^, respectively, are used to find an affine transformation matrix to fit *M* to M^*^.

The third step is transforming both *M* and M^*^ (as well as *L* and L^*^) to a frontal orientation with the forehead titled back by 10 degrees relative to the vertical axis, then transforming the representation to a cylindrical coordinate system (ρ, ϕ, *z*), where ρ, ϕ, and *z* represent the radial, the azimuth, and the height, respectively.

The fourth step is to warp *M* to M^*^ using the fiducial points *L* and L^*^ as control points. *L* and L^*^ are used to create a deformation function that warps *M* to M^*^. This study used the Thin-Plate Spline method [36], which minimizes a bending energy (or distortion) while maximizing the fit of *M* to M^*^, to compute the deformation function. The resulting deformation function was used to warp *M*.

The last step is to fully reproduce the given face model M^*^ using the set of 3D vertices associated with the reference face model *M.* This is done by linearly interpolating ρ for each point (ϕ, *z*) of *M* using the values (ρ, ϕ, *z*) of M^*^ as interpolants. Likewise, the RGB color values at each vertex of *M* were interpolated using these of M^*^. After this step, full correspondence of the resulting reproduced faces can be automatically achieved as they are generated from the same reference face model *M* (Figure 2C). Note that some vertices in the face can have the same ϕ and *z* value to that of others. This mostly happens in the ear area. As our method is applied to the facial area only (after removing ear area as described in the Eigen-disfigurement: surgically plausible disfigurement model section), the effect on this issue is not significant for our modeling technique.

#### Post-operative images with missing fiducial points

**Figure 3:**
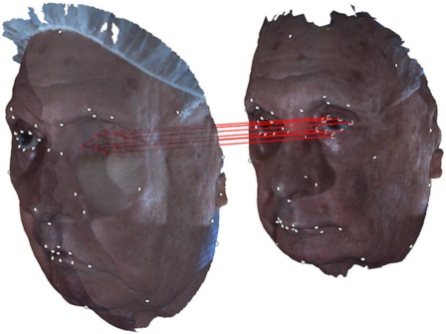
**Allocating missing fiducial points on the post-operative facial images.** Missing fiducial points on the post-operative facial image are allocated by projecting (red lines) the corresponding fiducial points of the pre-operative facial image of the same patient. White dots on both images, which indicate fiducial points unaffected by the surgery, are used to align the two images.

As previously mentioned, a patient may lose large portions of his/her face to disease and require a reconstructive surgery that substantially changes his/her facial morphology. In particular, he/she may need a reconstructive surgery in which a “flap”, a unit of tissue, usually comprised of skin, fat, muscle, bone or some combination of these types of tissue, is transplanted from another part of the body, such as the arm, leg, or trunk, and vascularized by an arterial input and venous output. For example, patients who underwent orbital exenteration followed by reconstructive surgery using an autologous flap are missing a substantial amount of the eye region of their faces and so do not have associated fiducial points available. To allocate fiducial points on the missing facial portion, we used the fiducial points of the same patient’s pre-operative image. To do so, we first aligned the pre-operative and post-operative images using the unaffected fiducial points. Then, the missing fiducial points can be found by projecting the corresponding fiducial points of the pre-operative image to the surface of the post-operative image (Figure 3).

#### Color normalization of 3D images

**Figure 4:**
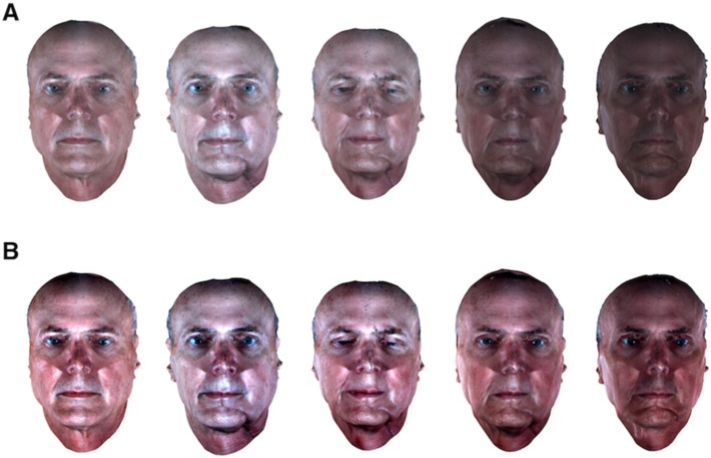
**Color normalization of 3D images. A**: Images of a patient showing high variation in color. **B**: Images of the same patient after contrast stretching each color channel, showing improvement of the color consistency.

**Figure 5:**
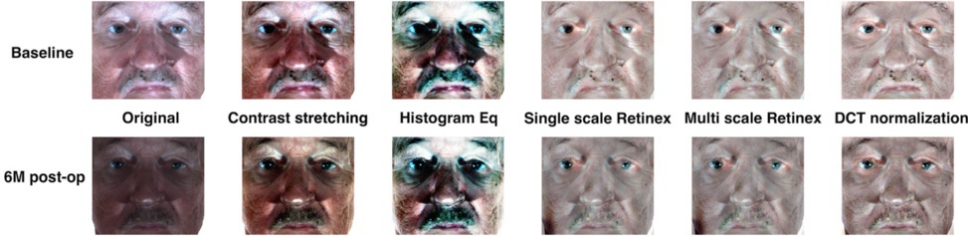
**Comparison of different color normalization techniques.** This figure provides visual comparison between different color normalization technique results. Although some illumination variations still exist, the contrast stretching compensated the color temperature difference among examples. Retinex algorithms (single and multi scale) and DCT based algorithm were able to compensate the brightness difference but lose variations in color, which is important for our application.

In many cases, the color statistics of 3D images of the same patient change over time; the changes include not only image brightness but also color temperature (Figure 4A). Such color changes may be viewed as artifacts that arise as the disfigurement model is developed. To reduce such color changes, we stretched the contrast of each color channel of the image such that only 1% of the data is saturated at low and high intensities of the image. Figure 4B shows the effectiveness of the contrast-stretching algorithm for the images of one patient over different time points. Although some illumination variations still exist, it compensated the color temperature difference among examples. There exist more sophisticated color alignment methods than contrast stretching (e.g., histogram equalization, Retinex algorithms [37,38], and DCT based algorithm [39]). However, visual inspection of the results of these algorithms on our data suggests that none of them is superior to the others (Figure 5). The Retinex algorithms and the DCT based algorithm were able to compensate for the brightness difference but lost variations in color, which is important for our application. Further studies of finding the best color alignment algorithms for this application are required, but it is out of the scope of this paper. In addition, we found contrast stretching to be simple and computationally efficient for this application.

### Eigen-disfigurement: surgically plausible disfigurement model

#### Defining a surgically plausible disfigurement model

Facial reconstruction for facial cancer patients cannot be achieved by a single operation. Multiple surgical operations are typically required until the patients complete the facial reconstruction. The best reconstruction strategy for each facial cancer patient is highly personalized since cancer can happen anywhere on the face, resulting in different reconstruction outcomes. Thus, this study focuses on modeling the unique disfigurement of each patient, and learning how such disfigurements change over the reconstruction process using a statistical modeling technique. It should be noted that patients can have more than one disfigurement; hence, we model each of them separately.

Let *F* be the 3D surface of the face. *F* consists of two components: 1) a structural component

1 $$ s=\left({x}_1,{y}_1,{z}_1,{x}_2,{y}_2,{z}_2,....,{x}_n,{y}_n,{z}_n\right)\in {\Re}^{3n} $$

where x, y, and z are the coordinates of the vertices of the 3D facial image, and 2) a textural component

2 $$ t=\left({r}_1,{g}_1,{b}_1,{r}_2,{g}_2,{b}_2,\dots, {r}_n,{g}_n,{b}_n\right)\in {\Re}^{3n} $$

where r, g, and b represent the red, green, blue color components at the vertices of the 3D facial image.

Then, define the surgically plausible disfigurement model to be a function that alters the given face *F* to the simulated one $\tilde{F}$:

3 $$ D\left(F,i,\lambda \right)=\left[\begin{array}{c}\hfill {D}_s\left(s,i,\lambda \right)\hfill \\ {}\hfill {D}_t\left(t,i,\lambda \right)\hfill \end{array}\right]=\left[\begin{array}{c}\hfill \tilde{s}\hfill \\ {}\hfill \tilde{t}\hfill \end{array}\right]=\tilde{F} $$

where *i* and *λ* are parameters that change the type (and therefore the location) and the degree of the disfigurement, respectively. The index *i* indicates the different types of disfigurements.

**Figure 6:**
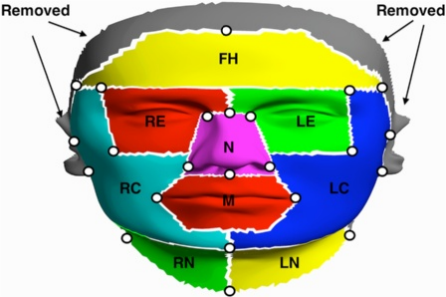
**Nine facial segments used in this study.** This figure illustrates a total of 9 facial segments (i.e., ROI) used in this study. The list of segments is: forehead (FH), right & left eye (RE & LE), nose (N), right & left cheek (RC & LC), mouth (M), right & left neck (RN & LN). Other areas were removed before further processing. A subset of 61 fiducial points (white dots) is used to determine the ROIs.

To take the local characteristics of facial disfigurements into account, we restrict our model to be learned and applied within specific facial regions of interest (ROIs): the forehead, the eyes (left and right), the nose, the cheeks (left and right), the mouth, the chin, and the neck (left and right). These 9 ROIs in total are depicted in Figure 6. We used a subset of the fiducial points (white dots in Figure 6) to determine the ROIs. The selection of the facial segment is based on a typical location where a given surgical treatment for facial cancer might cause facial disfigurement.

Now define the set *φ*_*i*_ = {*v*|*v* ∈ *F*} consisting of one or combinations of the aforementioned 9 ROIs, which is assumed to be affected by the *i*th disfigurement. Then the disfigurement model for the *i*th disfigurement can be further formulated as:

4 $$ {D}_s\left(s,i,\lambda \right)=\left\{\begin{array}{lll}\tilde{s}\hfill & if\hfill & v\in {\varphi}_i\hfill \\ {}s\hfill & if\hfill & v\notin {\varphi}_i\hfill \end{array},\kern1em {D}_t\left(t,i,\lambda \right)=\left\{\begin{array}{lll}\tilde{t}\hfill & if\hfill & v\in {\varphi}_i\hfill \\ {}t\hfill & if\hfill & v\notin {\varphi}_i\hfill \end{array}\right.\right. $$

where *v* are vertices in an target face *F*. Further define $\tilde{s}$ and $\tilde{t}$ as the results of stitching functions *f*_*s*_ and *f*_*t*_:

5 $$ {f}_s\left(s,\widehat{s}\right)=\tilde{s},\kern0.5em {f}_t\left(t,\widehat{t}\right)=\tilde{t}, $$

where *ŝ* and $\widehat{t}$ denote the structural and textural disfigurements learned from the patient images, respectively. Thus, the surgically plausible disfigurement model is a function that stitches the learned disfigurement within the corresponding ROI of the target face.

#### Eigen-disfigurement

As a first step toward developing the surgically plausible disfigurement model, we next describe how to learn the structural and textural disfigurement *ŝ* and $\widehat{t}$ from the patient images.

We utilized a common dimension reduction technique, PCA, to capture the *ŝ* and $\widehat{t}$ on patients’ faces. This is based on the fact that the appearance of the disfigured areas of patients’ faces will show high variations across his/her reconstruction process, since a facial disfigurement may imply major structural and textural changes on the face. Thus, we hypothesize that eigenvectors found from the faces of the same patient across the reconstruction process can capture for his/her facial disfigurement. We call these eigenvectors *Eigen-disfigurements* and used them to model *ŝ* and $\widehat{t}$.

Let *s*_*ij*_ be the structural face component of the patient exhibiting the *i*th type of disfigurement at the *j*th temporal moment of the reconstruction process. The variable *j* is an integer falling in the range 0 to *p,* where 0 represents the pre-operative visit, and *p* indicates the last post-operative visit. We compute the sample mean ${\overline{S}}_i$ of the shape components of a single patient with the *i*th type of disfigurement at different time instants, i.e., ${\overline{S}}_i={\displaystyle \sum_{j=0}^p{S}_{ij}}$. We can obtain the structural eigen-disfigurement *u*_*ik*_ of the patient’s face by computing the eigenvector of the covariance matrix given as

6 $$ Q=\frac{1}{p}{\displaystyle \sum_{{}^{{}_{j=1}}}^{{}_{{}^p}}{\Phi}_{ij}{\Phi}_{ij}^T,} $$

where ${\Phi}_{ij}={s}_{ij}-{\overline{S}}_i$. Since solving *Q*_*i*_ directly is infeasible, we first obtain the eigenvectors *û*_*k*_ of *Q*^*T*^, then compute the structural eigen-disfigurement

7 $$ {u}_{ik}={\displaystyle \sum_{j=1}^p{\sigma}_{kj}{\Phi}_{ij},k=1,\dots, p.} $$

The textural eigen-disfigurement *v*_*ik*_ of the patients’ face can be obtained similarly.

**Figure 7:**
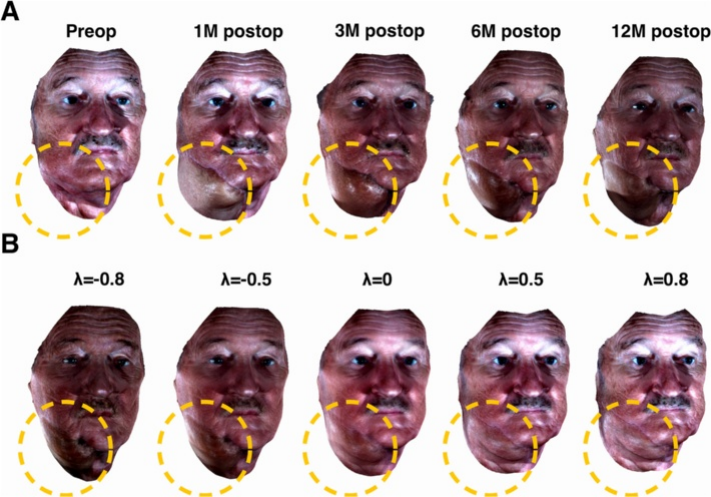
**Illustration of the concept of our Eigen-disfigurement model. A** shows the longitudinal changes of a patient who underwent reconstructive surgery on his right mandible and neck area (highlighted by yellow dashed circle). As shown, major structural and textural changes occur in the reconstructed area. **B** shows images of the same patient with varying degrees (i.e., *λ* values) along the direction of the first principal component. As the *λ* value deviates from 0, the degree of disfigurement increases. Specifically, as its value deviates towards −1, the texture/color of the disfigured region deviates (i.e., darker) from that of the typical healthy face. Moreover, as its value deviates towards 1, the structure of the disfigured region deviates from that of the typical healthy face. Thus the first principal component was sufficient to capture the disfigurement of the patient.

Once both the structural and the textural eigen-disfigurements are found, we can model *ŝ* and $\widehat{t}.$ Since the disfigurement is the major change in the face, the first few eigen-disfigurements should capture such change. We assumed that the first eigen-disfigurement is sufficient to capture the facial disfigurement. In fact, the first eigen-disfigurements (for both structural and textural disfigurement) are responsible for 50% of the total variation found from each patient’s data. Hence, the structural and textural disfigurements *ŝ* and $\widehat{t}$ for the *i*th disfigurement are

8 $$ \begin{array}{l}\widehat{s}={\overline{S}}_i+\lambda \cdot {u}_{ik}\hfill \\ {}\widehat{t}={\overline{T}}_i+\lambda \cdot {v}_{ik}\hfill \end{array} with\;v\in {\varphi}_i,-1\le \lambda \le 1, and\;k=1, $$

where *λ* is a variable that modifies the degree of disfigurement and (*u*_*ik*_, *v*_*ik*_)|_*k* = 1_ refers to the first eigen-disfigurement (having the largest eigen-value). Note that we can assign different parameters to control the structural and textural components separately and many face synthesis systems allow users to do so. However, this is not appropriate for simulating facial disfigurements of facial cancer patients. Surgical actions or radiation therapies affect both the structural and textural component of the face, and therefore, we need to consider them simultaneously. We also found statistically significant correlations between structural changes and textural changes arising from reconstruction surgery [40], which support our rationale. Figure 7 illustrates the concept of our eigen-disfigurement model; it captures the disfigurement from the patient’s longitudinal images.

### Stitching a surgically plausible disfigurement on a target face

We have now defined all of the parameters of the disfigurement model. Given proper stitching functions *f*_*s*_ and *f*_*t*_, we can simulate disfigurements of varying types, locations, and severities by adjusting the parameters *i* and *λ*.

The stitching functions should satisfy two conditions: 1) the simulated ROI should be smoothly connected to its boundary, and 2) the simulated ROI should capture the key characteristics of the learned disfigurement. We solved the problem by finding the interpolation functions that best fit the pre-defined guidance vector field from the boundary, thereby reconstructing the simulated structural and textural components within the ROI of the target face. We let the gradients of the learned disfigurements (∇*ŝ* and $\nabla \widehat{t}$) be the guidance vector fields. The formulation of the above problem is identical to that of the seamless-cloning feature of Poisson Image Editing [41], which was developed for 2D image editing, whereas our application is directed towards 3D surface images.

For each *i*th disfigurement, let ∂*φ*_*i*_ be the boundary of *φ*_*i*_ and let *f*_s_^*^ and *f*_t_^*^ be the known functions that determines the structural and textural components of the given face *F* excluding the *φ*_*i*_, respectively. Also let *α*_*s*_ and *α*_*t*_ be vector fields that guide the corresponding interpolation functions *f*_*s*_ and *f*_*t*_, to display the key characteristics of the disfigurement.

Considering the structural component first (as the textural component can be computed similarly), the function *f*_*s*_ achieving the above two conditions can be found by solving the following minimization problem:

9 $$ \underset{fs}{ \min }{\displaystyle \underset{\varphi_i}{\iint}\left|\nabla {f}_s-{a}_s\left|{}^2\; with\;{f}_s\left|{}_{\partial \varphi i}={f}_s^{*}\right.\left|{}_{\partial \varphi i}\right.\right.\right.} $$

where ∇ represents the gradient operator. Its solution can be obtained by solving the following Poisson equation with Dirichlet boundary condition:

10 $$ \Delta {f}_s=div\left({\alpha}_s\right)\left|{}_{\varphi i}\; with\;{f}_s\left|{}_{\partial \varphi i}={f}_s^{*}\right.\left|{}_{\partial \varphi i}\right.\right. $$

where ∆ and *div*(⋅) represent the Laplacian operator and divergence, respectively.

To apply the above minimization to our application, we discretized the problem and solved it numerically. Let Ω be the set of vertices that defines each triangulated mesh on the facial surface image. Further denote (*a*, *b*) to be the vertex pair defined by the triangulation set Ω. Then we can define the weight matrix

11 $$ {W}_{a,b}=\left\{\begin{array}{ll}1\hfill & if\left(a,b\right)\in \varOmega \hfill \\ {}0\hfill & otherwise\hfill \end{array}\right., $$

which indicates adjacencies between vertices. Let *τ*_*a*_ = ∑_*b*_*W*_*a*,*b*_ be a connectivity weight vector, which counts the number of edges connected to the vertex *a*. Then the Laplacian operator can be computed in matrix form as follows,

12 $$ L=\Gamma -W, $$

where Γ = *diag*(*τ*_1_, …, *τ*_*n*_).

As previously mentioned, we used the gradient of the learned disfigurement (∇*ŝ* and $\nabla \widehat{t}$) to guide the vector field (*α*_*s*_ and *α*_*t*_). Then, the Poisson equation (10) can be expressed as,

13 $$ \Delta {f}_s=\Delta \widehat{s}\; over\;{\varphi}_i,\; with\;{f}_s\left|{}_{\partial \varphi i}={f}_s^{*}\left|{}_{\partial \varphi i}\right.\right. $$

where it can be formulated as the following linear equations:

14 $$ \begin{array}{c}\hfill {\displaystyle \sum_{b=1}^m\left({L}_{a,b}\cdot {f}_s\left|{}_{v=b}\right.\right)={\displaystyle \sum_{b=1}^m\left({L}_{a,b}\cdot \widehat{s}\left|{}_{v=b}\right.\right),\mathrm{if}}\;}b\notin \partial {\varphi}_i\hfill \\ {}\hfill {f}_s{\left|{}_{v=b}={f}_s^{*}\right|}_{v=b},\mathrm{if}\;b\in \partial {\varphi}_i\hfill \end{array}, $$

where *m* is the total number of vertices in φ_*i,*_ and *f*_*s*_|_*v* = *b*_ and *ŝ*|_*v* = *b*_ refer to the structural information contained in *f*_*s*_ and *ŝ* at the vertex *v* = *b*, respectively*.*

**Figure 8:**
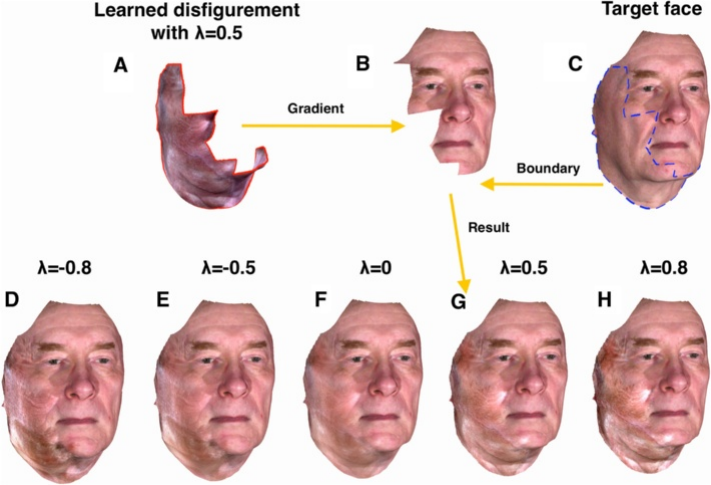
**Illustration of how the stitching function works to create simulated faces with disfigurements.** The stitching function finds the interpolation functions that follow the gradient of the learned disfigurement (gradient of structural and textural part inside of red boundary line in **A**) from the boundary of the target face (blue dashed line in **C**). Sub-figures **D**-**H** are simulation results for varying degrees of disfigurement on the target face **B**. It may be seen that the stitching functions *f*_*s*_ and *f*_*t*_ smoothly connect the learned disfigurements of varying degrees to the target face using the unknown boundary of the ROI of the target face and gradient of the learned disfigurement.

The above linear equation can be solved using an iterative algorithm. We used the biconjugate gradient method [42] to solve the above sparse equation, i.e., to compute *f*_*s*_ for each of the x, y, and z components separately. In all cases, the least square solutions are found within 1000 iterations. Figure 8 shows how the stitching function works; it smoothly connects the learned disfigurement of varying degree to the target face within the ROI of the target face using gradient information from the learned disfigurement.

### Evaluation strategy

#### Evaluation of preprocessing step

The disfigurement model that this study proposes is based on 3D facial surface images of patients reproduced from original 3D images, using the model mannequin face to achieve correspondence across images. Thus, a reliable and accurate algorithm to reproduce the 3D faces with full correspondence is necessary.

**Figure 9:**
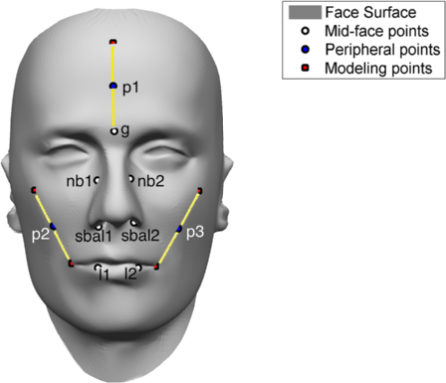
**Location of validation fiducial points.** A total of 10 validation fiducial points were used to evaluate the pre-processing step. Among those, 7 were located on the mid-face area (white dots) and the other 3 were located on the periphery (blue dots). For those points on periphery, we used the surface point on the middle between two existing fiducial points, which were used in the pre-processing step (red dots, annotated as modeling points). Yellow lines indicate what modeling points were used to obtain the peripheral validation fiducial points.

To evaluate the quality of the preprocessing step, we tested if fiducial points that were not used for the preprocessing step can be accurately retrieved, which is similar to the method described in [43]. First we placed the additional fiducial points on the model mannequin face and each of 3D facial surface images (both disfigured and non-disfigured set). We call these fiducial points as validation fiducial points. Then, we computed the error between the validation fiducial points of a given 3D facial surface image and those of its reproduced version from the model mannequin face. A total of 10 validation fiducial points were annotated and used for this analysis (Figure 9). Note that these validation fiducial points were not used for the preprocessing step. First 7 fiducial points (white dots in Figure 9) are based on the previous literatures (e.g., [24,34]), where mainly located in mid-face area. The other 3 fiducial points are in peripheral. Since there are less visible fiducial points in peripheral than mid-face area, we mathematically computed the location of these 3 fiducial points from the pre-existing fiducial points; we used the surface point on the middle between two pre-existing fiducial points. Euclidean error for the 10 additional fiducial points will be minimized as the algorithm effectively reproduces the given face with full correspondence to other faces.

#### Sensitivity to fiducial point allocation

We evaluated how sensitive the algorithm is to errors introduced by fiducial point allocation since such errors can affect the overall quality of the reproduced face. For this, we randomly selected one face pair from each dataset (disfigured and non-disfigured) and the preprocessing algorithm was reapplied after randomly scrambling the locations of the fiducial points. It was found that the maximum error was 1.49 *mm* when human raters annotated the fiducial points [35]. Next, we scrambled the location of each fiducial point (excluding additional fiducial points introduced in the previous chapter) by 1.5 – 3 *mm* in increments of 0.5 *mm.* We then repeated the error analysis as described in the previous section for each case to check the effect of the introduced perturbations in fiducial point allocation for the overall quality of the reproduced face. We excluded the 3 additional fiducial points in peripheral for this analysis as the scrambling process can perturb their locations. The aforementioned procedures were repeated 10 times to obtain summary statistics (e.g., average) of the above measures.

#### Evaluation of disfigurement model

The ultimate purpose of this study is to provide a new tool that allows us to understand human impressions of visible disfigurements while being able to control the location and level of the severity of disfigurement. Our goal is not to estimate physical properties of a reconstructive surgery outcome, but rather, to determine whether the resulting *simulated* disfigurement is plausible or not.

The best way to evaluate the visual plausibility of the *simulated* disfigurement is to obtain subjective opinions of medical professionals who have clinical experience in the treatment of patients with head and neck cancer. Thus, we conducted an observer study using 4 medical professionals under an approved IRB protocol from The University of Texas at Austin (Protocol ID of 2013-10-0065). The participating medical professionals included 2 plastic/reconstructive surgeons, 1 nurse, and 1 physician assistant (PA) employed at the Seton Medical Center in Austin, Texas, USA. All medical professionals provided informed consent (verbal) to participate the study. These medical professionals were not involved in the development of the disfigurement model. Here after we shall refer to these 4 medical professionals as observers.

##### Simulated image set for observer study

**Figure 10:**
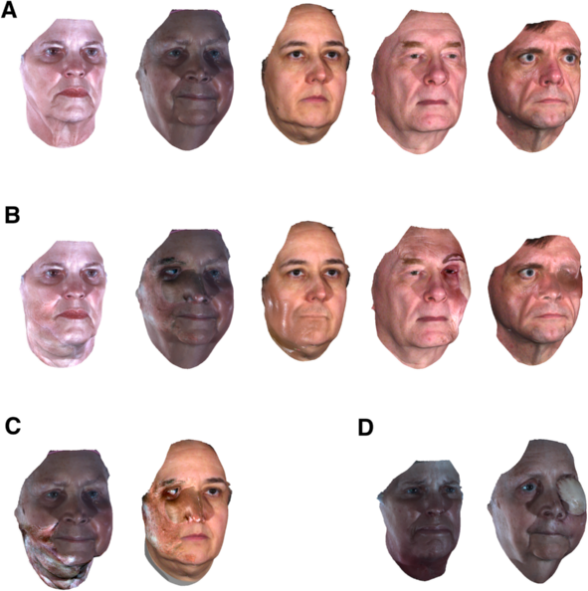
**Examples of simulated and real disfigurements.** In subfigure **A**, the first two images from the left are from the disfigured dataset while the others are from the non-disfigured dataset. From left to right, subfigure **B** shows: 1) disfigurement due to a flap on the left mandible and neck, 2) disfigurement due to a flap around the nose and eye area, 3) disfigurement due to a mandibulectomy scar on the mouth and neck, 4) disfigurement due to a flap on the right eye and forehead, and 5) disfigurement due to a flap on the right eye, respectively. Subfigure **C** shows implausible results created by exaggerating the degree of disfigurement. Their plausible versions are shown in the first two simulations in **B**. Subfigure **D** shows real disfigurements. The patients’ pre-operative faces are the first two faces in **A**.

We selected a total of five 3D facial images (3 female and 2 male, all non Hispanic/Latino White to match the major race/ethnic group in the disfigured set) as target faces for the simulation (Figure 10A). Among the 5 images, 2 were from the dataset of disfigured faces while 3 were from the dataset of non-disfigured faces. The 3 individuals from the non-disfigured dataset had ages typical of facial cancer patients (>45 old). After removing visually subtle disfigurements or disfigurements having similar shape and texture each other (1 mid-face and 3 periphery), we applied 13 disfigurements (the first 6 mid-face disfigurements and the first 7 peripheral disfigurements listed in Table 1) developed from our modeling technique on randomly selected male target faces. The same 13 disfigurements were also applied on randomly selected female target faces. For those 26 simulations, we fixed *λ* = 0.5 (Figure 10B). To test the observers’ responses to implausible results, we also included 4 implausible simulations (2 mid-face disfigurements and 2 peripheral disfigurements) by exaggerating the degree of disfigurement by setting *λ* = 1.3 (Figure 10C). In addition, for comparison, we included two 3D facial images of patients having real disfigurements (Figure 10D). These images were not used to develop our disfigurement model. Therefore, a total of thirty two 3D facial images were prepared for evaluation of the proposed disfigurement modeling technique.

##### Observer study setup

Each 3D simulated face was displayed on a typical personal computer screen. Each 3D face was rendered on the screen and observers were allowed to evaluate the facial appearance fully by rotating the face and zooming in or out of the 3D scene.

**Figure 11:**
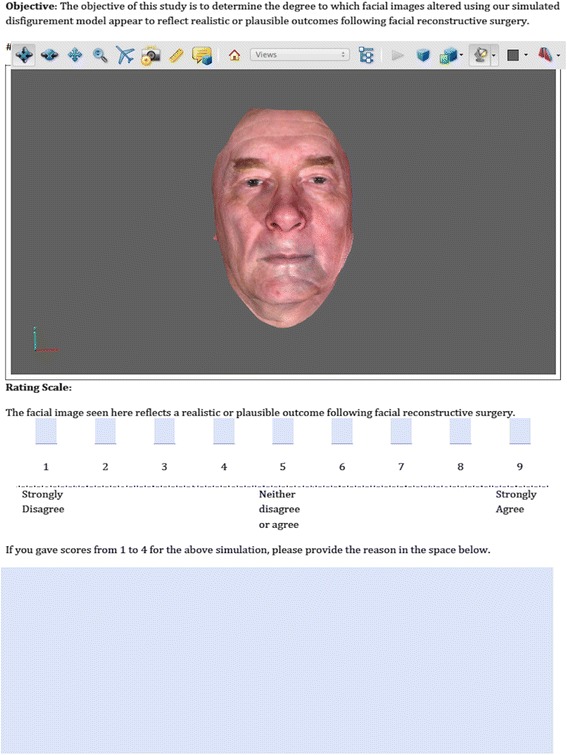
**Screen layout of the evaluation study.** Observers were allowed to examine the given stimuli fully by rotating the rendered 3D faces and zooming in or out of the 3D scene.

After the review, they were asked to rate the plausibility of the simulation result using a 9-point Likert scale. A value of 1 indicates that they strongly disagreed that the depicted disfigurement could be seen as an outcome following facial reconstructive surgery, while a value of 9 indicates that they strongly agreed that the depicted disfigurement could be seen as a reconstruction outcome. The duration of the study was approximately 40 minutes for each observer. Figure 11 shows the layout of the experiment for this study.

##### Statistical analysis for observer study

We performed a statistical modeling of the observers’ ratings to investigate the plausibility of different types of facial disfigurement simulations. In addition to the simulation type, gender of target faces was included as a covariate since previous literatures suggest that there may an inherent bias in observer’s perception on facial lesions (e.g., [44]). Moreover, the observers’ criteria of assessing the plausibility of the facial disfigurement are expected to show some variability. Thus, we used a mixed model to properly model factors affecting observers’ ratings as well as the inter-observer variability. Among many variations of mixed models, we utilized a cumulative link mixed model as observer’s ratings are ordinal in nature:

15 $$ \log \mathrm{it}\left(P\left({r}_i\le j\right)\right)={\theta}_j+\beta {X}_i+Ob{s}_i,i=1,\dots, 128,j=1,\dots, 8 $$

where *r*, *X*, and *Obs* are the observers’ ratings, the fixed effects, and the random effects, respectively. In addition, *i* indexes all ratings, *β* corresponds to the coefficient associated with *X*, and *θ*_*j*_ is a threshold value for *j*th Likert scale level. This model accounts for the cumulative probability distribution of the *i*th rating being in the *j*th Likert scale level. The simulation types (mid-face, periphery, real, and exaggerated) and gender of each target face are considered as the fixed effects *X*_*i*_. The inter-observer variability is modeled as random effects $Ob{s}_i\sim N\left(0,{\sigma}_{Obs}^2\right).$ Note that we did not stratify the real and exaggerated simulation samples further to create additional (sub) types due to the limited number of available samples in both cases.

The questions that we are interested in are: 1) whether there is any difference in observer-rated plausibility between the simulated faces, the real patient faces, and the exaggerated faces, and 2) whether the plausibility ratings on simulation results are affected by the gender of the target face. This study used the ordinal package of the R v.3.0.3 [45] to build a cumulative link mixed model and answer the above questions.

## Results

### Evaluation of preprocessing step

The results show that the preprocessing step effectively reproduced the given face using the reference mannequin model (Table 2). For both datasets, the averaged error for each validation fiducial points ranged from 1.2 *mm* to 4.4 *mm*. The average error for the points around nose (nb1 and nb2 in Figure 9) and the peripheral point on forehead (p1 in Figure 9) were relatively higher than for the other points (which ranged from 3.2 *mm* to 4.4 *mm*). These validation fiducial points have less neighboring fiducial points than the other validation fiducial points. This means they have more freedom to move away from the point where it should be. However, the amount of error was still small (less than 5 *mm*) compared with the degree of morphological change due to the reconstructive surgery.

**Table 2:** **Error between the pre-processed face and the given face for validation fiducial points**

**Validation fiducial points**	**Error (** ***mm*** **)**			
	**Disfigured set**		**Non-disfigured set**	
	**Mean**	**Std**	**Mean**	**Std**
g	1.2	0.7	1.4	0.7
nb1	3.5	2	4.2	2.2
nb2	4.4	2.2	3	1.7
sbal1	2.8	1.3	2.6	1.2
sbal2	3	1.6	3.4	1.7
l1	2.2	1.2	2	1.1
l2	3	1.4	3.7	1.5
p1	3.2	1.7	2.9	1.7
p2	2.3	1.3	2.1	1.1
p3	2	1.3	1.7	0.9

### Evaluation of fiducial point allocation sensitivity

The results show that there was no significant effect on the error introduced by the fiducial points allocation (Table 3). Although the error increased with the amount of perturbation introduced, the increased amounts are limited (mostly less than 5 *mm*). Thus, the effect of errors in fiducial point allocation on the overall quality of the preprocessed faces and the subsequent disfigurement models was minimal.

**Table 3:** **Evaluation results for fiducial point allocation sensitivity analysis**

		**Mean error between the preprocessed face and the original face (** ***mm*** **)**				
***Perturbation error (mm)***		***0***	***1.5***	***2***	***2.5***	***3***
***Validation fiducial points***						
Disfigured sample	g	2.2	2.8	2.4	3.3	4.2
	nb1	4	3.5	3	3.4	5.2
	nb2	3.8	4.3	4.2	4.2	4.7
	sbal1	1.9	2.1	1.8	2.2	3.2
	sbal2	2.7	3	3.6	3.3	4.5
	l1	0.9	1.3	1.8	2	2.1
	l2	1.5	1.4	1.6	1.7	3.3
Non-disfigured sample	g	1.6	1.9	2.5	2.3	2.6
	nb1	2.7	2.5	2.4	3.7	3.7
	nb2	5.4	6	6	5.7	7
	sbal1	2.7	3	3	3.6	4.3
	sbal2	1.2	1.7	1.9	2.1	2.3
	l1	2.3	2.2	2.1	3	2.8
	l2	2.4	2.8	2.1	2.2	2.7

### Observer evaluation of disfigurement

The test for differences in gender shows that there was no statistically significant gender effect on observer’s plausibility ratings (p-value = 0.64) when considering different simulation types (Table 4). Similarly, the test for differences between the real samples and the other simulation types indicate that there was no statistically significant difference in observer plausibility ratings (p-value = 0.08) between the real samples and the simulations of peripheral disfigurements when considering gender. However, we found opposite results (p-values < 0.001) for mid-face and exaggerated simulated disfigurements. This demonstrates that our modeling technique was effective when simulating peripheral disfigurements. However, mid-face simulations were not rated as similar to the real samples.

**Table 4:** **Cumulative link mixed model analysis results**

**Fixed-effects**		**Coefficient**	**Standard error**	***p*** **-value**
Simulation type	Mid-face	−2.99	0.79	<0.001
	Peripheral	−1.31	0.75	0.08
	Exaggerated	−7.37	1.09	<0.0001
Gender	Female	−0.15	0.33	0.64
Random-effects		Variance	Standard deviation	
Observer (Intercept)		0.68	0.83	N/A

Final cumulative link mixed model estimates for each fixed, and random effect variable, as well as the result of testing for difference in observer ratings for simulation types and gender. For the simulations, the tests for difference in ratings were against real disfigurement samples. For gender, the test was against male target face samples.

**Figure 12:**
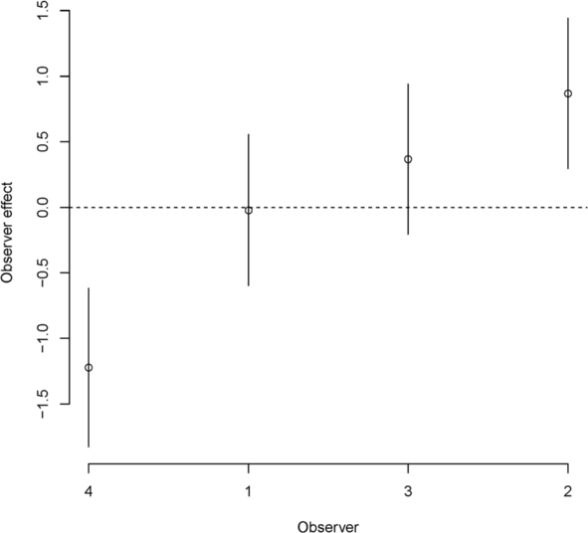
**Observer effects via conditional modes with 95% confidence intervals based on the conditional variance.** This figure shows that the fourth observer gave the lowest plausibility ratings, while the second observer gave the highest plausibility ratings. These variations on ratings may indicate that observers perceive the plausibility of simulation samples differently.

In addition, we evaluated the observer effects by conducting a likelihood ratio test between the original cumulative link mixed model and an additional cumulative link model without observer effects. The chi-squared test on the likelihood ratio showed significant difference between two models (χ^2^ = 14.88, df = 1, p-value <0.001), which indicates that the observer-level random effects are significant. We further evaluated the observer effects by estimating their conditional modes with 95% confidence intervals based on the conditional variance (Figure 12). The fourth observer gave the lowest plausibility ratings to simulations, while the second observer gave the highest plausibility ratings. These results indicate that observers perceive the plausibility of simulation samples differently.

To evaluate the trend of observer ratings in detail, we computed summary statistics for each simulation type, and for each gender. As expected, the real disfigurement samples were rated higher (group median: 7.25) than the others (Table 5). In addition, the exaggerated disfigurement simulations were rated lower (group median: 1.75) than the others. The mid-face (group median: 5.5) and peripheral (group median 6.5) disfigurement examples were rated between the ratings of the real and exaggerated samples. Although there are some exceptions, most of simulated disfigurements received median ratings above 5, which means the observers were prone to believe that those simulations were plausible facial cancer reconstruction outcomes. Two mid-face simulations (M1 and M4 in Table 5) were rated as implausible results. The disfigured regions of patients M1 and M4 are wider than on the 4 patients with mid-face disfigurement. In fact, the disfigured region of patient M6 is smaller than the others and its simulation on the target faces got high ratings (especially on male target). This indicates that the observers perceive a wider and larger disfigurement simulation as less plausible.

**Table 5:** **Summary statistics of the medical professionals’ ratings on simulated, real, and exaggerated disfigurement**

**Types**	**Location/gender of target face**	**Disfigurement source**	**Medical professionals’ ratings (N = 4)**				
			***Median***	***MAD***	***Min***	***Max***	***Overall***
*Simulated (λ = 0.5 | N = 26)*	*Mid-face female target (N = 6)*	M1	2.5	0.5	2	5	5.5
		M2	6	0.5	5	7	
		M3	5.5	1	4	8	
		M4	4.5	0.5	3	5	
		M5	6	0.5	4	7	
		M6	5.5	1.5	3	7	
	*Mid-face male target (N = 6)*	M1	4	1.5	2	7	5
		M2	5	1.5	2	7	
		M3	5	0	3	5	
		M4	4.5	0.5	4	6	
		M5	5.5	2	3	8	
		M6	7	0.5	4	8	
	*Peripheral female target (N = 7)*	P1	7.5	0.5	7	9	6.5
		P2	6.5	0.5	6	8	
		P3	6.5	0.5	5	7	
		P4	6	1	2	7	
		P5	6.5	0.5	4	7	
		P6	6	1	5	8	
		P7	7	0.5	6	8	
	*Peripheral male target (N = 7)*	P1	7	0	7	9	6.5
		P2	6.5	1	4	8	
		P3	7	0.5	5	8	
		P4	6	1	4	7	
		P5	7	0.5	5	8	
		P6	6.5	1	3	8	
		P7	6	1	6	8	
*Real (N = 2)*	*Mid-face*	N/A	8	0.5	7	9	7.25
	*Peripheral*		6.5	1	5	8	
*Exaggerated (λ = 1.3 | N = 4)*	*Mid-face (N = 2)*	M1	2	0.5	1	4	1.75
		M3	1.5	0.5	1	3	
	*Peripheral (N = 2)*	P2	1	0	1	2	
		P3	2	0.5	1	7	

MAD refers to median absolute deviation, which is computed as the median of the absolute deviations from the median of the data.

## Discussion

We proposed a new strategy to learn facial disfigurements from real patient data that persist after ablative and reconstructive surgery of facial cancers. We subsequently used the gathered data to simulate such disfigurements on the faces of other individuals by a modeling process. Unlike previous studies investigating how human perceive facial disfigurements, this study utilized modeling techniques that provide control over the type, location, and degree of disfigurement, enabling controlled and systematic experiments on the human perception of disfigurements.

From the 3D surface facial images of patients with facial disfigurement, the algorithm first reproduces each face from a facial mannequin model to establish full correspondence between the faces. Using the reproduced faces, an algorithm derived from the model learns the longitudinal structural and textural changes (disfigurements) on each patient’s face over the course of the treatment. This algorithm enables plausible simulations by smoothly imposing the learned disfigurements on the corresponding part of the faces of others.

Quantitative evaluation of the reproduced faces showed that the algorithm was able to effectively reproduce each given face using a facial mannequin model. We also showed that human error during fiducial point allocation could introduce errors in modeling. However, these errors were very small (mostly less than 5 *mm*) as compared to structural changes that patients can experience during treatment.

To show that the proposed modeling strategy can be used to investigate how humans perceive disfigurement, we evaluated the plausibility of the *simulated* examples using panel ratings of experienced medical professionals, blind to the source of each image. We prepared a total of 32 facial images for evaluation. Four types of samples were prepared: 1) mid-face, 2) periphery, 3) real, and 4) exaggerated. Based on statistical analysis of the observer ratings, our disfigurement modeling scheme was able to create simulation results with plausibility ratings similar to real disfigurement samples for periphery disfigurements. While mid-face simulations were rated as lower than real and periphery samples, in most cases these also were rated as plausible reconstructive surgery outcomes.

We found a significant observer-level random effect in plausibility ratings. Moreover, we found that observers tended to rate mid-face simulations with wider affected regions as lower than those with smaller affected regions. This may indicate that each observer has a different threshold of plausibility. In the simulations, we fixed the degree of disfigurement *λ* = 0.5 for both mid-face and peripheral disfigurements. It is possible that the observers may have perceived such a fixed degree of disfigurement differently on the different facial areas, thereby affecting his/her final ratings. This could explain why the mid-face simulations were rated lower than peripheral simulations. It is also possible that setting *λ* = 0.5 resulted in mid-face disfigurements that were too large, especially for disfigurement with wide affected regions. Further studies with varying *λ* values will be required to confirm this. However, the variation found in the observer ratings on each simulation is strong motivation to create a model to study human perception of disfigurement.

One limitation of this study is that the algorithm may decide that an error having greater variation than a real disfigurement is also a disfigurement. Conversely, the algorithm may ignore minimal disfigurements with less variation than natural longitudinal variations of a patients’ face morphology. This is due to the fact that our modeling technique utilizes PCA to capture longitudinal structural and textural changes (disfigurements) of a patient during treatment. Since PCA only aligns the data in terms of the amount of variance found in it, any error causing high variation could be detected as disfigurement. Specifically, large illumination changes of one image relative to another of the same patient could mislead our modeling algorithm to regard such illumination error as disfigurement. However, such illumination changes could be controlled at the acquisition stage by applying a rigorous calibration step on 3D image acquisition and by maintaining the ambient light conditions. Visually minimal disfigurements usually occur when the oncological and reconstructive surgeries were conducted internally. In such cases, many disfigurements are visually subtle or even not superficially visible. Even if the algorithm extracts such subtle disfigurements, it may not be useful to develop a disfigurement model from it since it may not be noticeable to a human observer. In addition, pre-existing facial characteristics of patients such as facial wrinkles or surgical scar (e.g., Figures 1 and 8) can cause an artifact in our simulation results. Since the pre-existing characteristics do not show temporal changes, they can stay in DC component (or mean) of Eigen-disfigurement, which can cause a visual artifact. However, we can prevent this artifact by removing it before building Eigen-disfigurement; one can use the concealment feature of Poisson Image Editing [41] for this.

The ultimate goal of this study was to provide models that can simulate surgically plausible disfigurements with control of the location and degree of the disfigurement. In this respect, the obvious clinical application of our modeling method is to investigate how humans perceive disfigurements by varying the location and degree of disfigurement severity. Moreover, our model can be used for patient consultation. Care providers (e.g., surgeons or psychologists) could use an image showing the *simulated* disfigurement of a patient who will undergo certain oncological and reconstructive surgery for facial cancer for surgical planning, or patient education (i.e., helping him/her to understand and cope with possible changes to his/her face that are expected due to surgery).

Future applications of this study include: 1) conducting an additional human observer study using medical professionals to investigate inter- and intra-rater variability and to find appropriate ranges of disfigurement levels as we found variations in their plausibility ratings; 2) conducting a human observer study to determine how the type, location, and severity of disfigurement affects human perception. This will require observers that are unfamiliar with facial cancer patient deformities; 3) testing/validating existing algorithms or further developing it to locate fiducial points automatically on 3D faces of patients with facial disfigurements; and 4) investigating how state-of-the-art face recognition algorithms perform on faces with simulated disfigurement. The first task is needed to further refine our disfigurement models for future studies. The results of the second task may foster a deeper understanding of human perception of disfigured faces, which can be used to help patients with such disfigurements to psychosocially adjust to live with those conditions. The results of third task could facilitate the overall processing efficiency of the disfigurement modeling process. The last task may prove highly interesting for developing security and defense applications. Since most previous studies have focused on the healthy population instead of patients with facial disfigurements, even state-of-the-art face recognition algorithms may not succeed on individuals with facial impairments. By using the proposed disfigurement models, we could create different types of disfigurements at various locations on a face. Accordingly, we could be able to systematically validate existing algorithms and help other researchers develop optimal methods robust to such facial variations.

## Conclusion

This study introduced a framework to learn and extract facial disfigurements from real patient data that persist after oncologic and reconstructive surgery of facial cancers, and subsequently to model and apply such disfigurements on novel faces with a high degree of control of disfigurement types. The modeling technique was able to capture facial disfigurements and its simulation represents plausible outcomes of reconstructive surgery for facial cancers, especially for disfigurements on the facial periphery. In the future, the framework introduced by this study could be used to understand how human perceive facial disfigurements systematically by varying its type and severity.

## Additional file

Additional file 1:
**This table shows the details of reconstruction procedures for each patient.**
